# An Account of Diseases in an Eastern District of London from April 20, to May 20, 1809

**Published:** 1809-06

**Authors:** 


### *An Account of Diseases in an Eastern District of London from April 20, to May 20, 1809.*

| | |
|---|---|
| **ACUTE DISEASES.** | |
| Typhus | 2 |
| Peripneumonia Notha | 5 |
| Rheumatismus Acutus | 3 |
| **CHRONIC DISEASES.** | |
| Dyspnœa | 5 |
| Tussis cum Dyspnœa | 12 |
| Tussis | 7 |
| Hydrops Pectoris | 4 |
| Palpitatio | 2 |
| Ascites | 3 |
| Anasarca | 5 |
| Enterodynia | 3 |
| Dysuria | 5 |
| Scrophula | 4 |
| Cephalalgia | 6 |
| Vertigo | 3 |
| Paralysis | 2 |
| Hysteria | 4 |
| Fluor Albus | 6 |
| Amenorrhœa | 3 |
| Rheumatismus Chronicus | 7 |
| **PUERPERAL DISEASES.** | |
| Dolor post Partum | 6 |
| Abscessus Mammæ | 2 |
| Mastodynia | 4 |
| **INFANTILE DISEASES.** | |
| Pertussis | 3 |
| Vermes | 4 |
| Scrophula | 2 |
| Ophthalmia | 3 |

In a former monthly report of diseases it was remarked, that some faint appearance of hydropic symptoms had occurred amongst patients who had been attacked by different diseases of the chest. Too frequently, indeed, does such a termination occur; and those who are congratulated upon their having escaped from an alarming inflammation of the lungs, or some other threatening disease, become the objects of commiseration on account of the appearance of some other symptoms, which, though not so immediately, yet ultimately prove fatal to life. This has been exemplified in several instances referred to in former lists. After patients had so far recovered as to return to their their usual occupations, and to resume their former regimen and habits, they have perceived the gradual approach of swelling and enlargement of the feet and ancles, particularly in the evening. After some time the face and upper extremities appear in a state of distention in the morning. The urine is secreted in small quantities, and a fluid is collected in different parts of the cellular membrane, till at last a complete anasarca is formed. The most alarming symptom however, which supervenes, is the great difficulty of breathing, and which gives to the disease the character of hydrops pectoris. The patient sometimes awakes very suddenly in the night with a sense of suffocation; the pulse becomes irregular, a palpitation of the heart occurs; and with some or other of these symptoms he drags on a miserable existence, till at length the disease proceeds, sometimes more gradually, and at other times more suddenly, to a fatal termination.

